# Relationship Between a Vitamin D Genetic Risk Score and Autoantibodies Among First-Degree Relatives of Probands With Rheumatoid Arthritis and Systemic Lupus Erythematosus

**DOI:** 10.3389/fimmu.2022.881332

**Published:** 2022-06-03

**Authors:** Lauren A. Vanderlinden, Elizabeth A. Bemis, Jennifer Seifert, Joel M. Guthridge, Kendra A. Young, Mary Kristen Demoruelle, Marie Feser, Wade DeJager, Susan Macwana, Ted R. Mikuls, James R. O’Dell, Michael H. Weisman, Jane Buckner, Richard M. Keating, Patrick M. Gaffney, Jennifer A. Kelly, Carl D. Langefeld, Kevin D. Deane, Judith A. James, Vernon Michael Holers, Jill M. Norris

**Affiliations:** ^1^Colorado School of Public Health, University of Colorado Anschutz Medical Campus, Aurora, CO, United States; ^2^School of Medicine, University of Colorado Anschutz Medical Campus, Aurora, CO, United States; ^3^ Arthritis & Clinical Immunology Research Program, Oklahoma Medical Research Foundation, Oklahoma City, OK, United States; ^4^Division of Rheumatology and Immunology, University of Nebraska Medical Center and VA Nebraska-Western Iowa Health Care System, Omaha, NE, United States; ^5^Division of Rheumatology, Cedars-Sinai Medical Center, Los Angeles, CA, United States; ^6^Center for Translational Immunology, Benaroya Research Institute (BRI) at Virginia Mason, Seattle, WA, United States; ^7^Division of Rheumatology, Scripps Health, La Jolla, CA, United States; ^8^Department of Biostatistics and Data Science, Wake Forest School of Medicine, Winston Salem, NC, United States; ^9^Center for Precision Medicine, Wake Forest School of Medicine, Winston Salem, NC, United States

**Keywords:** vitamin D, autoantibody positive (aAb+), autoantibody negative (aAb-), rheumatoid arthritis (RA), systemic lupus erythematosus (SLE), genetic risk score (GRS)

## Abstract

**Objective:**

Higher 25-hydroxyvitamin D (25(OH)D) levels have been associated with reduced risk for autoimmune diseases and are influenced by vitamin D metabolism genes. We estimated genetically-determined vitamin D levels by calculating a genetic risk score (GRS) and investigated whether the vitamin D GRS was associated with the presence of autoantibodies related to rheumatoid arthritis (RA) and systemic lupus erythematosus (SLE) in those at increased risk for developing RA and SLE, respectively.

**Methods:**

In this cross-sectional study, we selected autoantibody positive (aAb+) and autoantibody negative (aAb-) individuals from the Studies of the Etiologies of Rheumatoid Arthritis (SERA), a cohort study of first-degree relatives (FDRs) of individuals with RA (189 RA aAb+, 181 RA aAb-), and the Lupus Family Registry and Repository (LFRR), a cohort study of FDRs of individuals with SLE (157 SLE aAb+, 185 SLE aAb-). Five SNPs known to be associated with serum 25(OH)D levels were analyzed individually as well as in a GRS: rs4588 (*GC*), rs12785878 (*NADSYN1*), rs10741657 (*CYP2R1*), rs6538691 (*AMDHD1*), and rs8018720 (*SEC23A*).

**Results:**

Both cohorts had similar demographic characteristics, with significantly older and a higher proportion of males in the aAb+ FDRs. The vitamin D GRS was inversely associated with RA aAb+ (OR = 0.85, 95% CI = 0.74-0.99), suggesting a possible protective factor for RA aAb positivity in FDRs of RA probands. The vitamin D GRS was not associated with SLE aAb+ in the LFRR (OR = 1.09, 95% CI = 0.94-1.27). The *SEC23A* SNP was associated with RA aAb+ in SERA (OR = 0.65, 95% CI = 0.43-0.99); this SNP was not associated with SLE aAb+ in LFRR (OR = 1.41, 95% CI = 0.90 – 2.19).

**Conclusion:**

Genes associated with vitamin D levels may play a protective role in the development of RA aAbs in FDRs of RA probands, perhaps through affecting lifelong vitamin D status. The GRS and the *SEC23A* SNP may be of interest for future investigation in pre-clinical RA. In contrast, these results do not support a similar association in SLE FDRs, suggesting other mechanisms involved in the relationship between vitamin D and SLE aAbs not assessed in this study.

## Introduction

Rheumatoid arthritis (RA) and systemic lupus erythematosus (SLE) are chronic inflammatory autoimmune diseases (ADs) thought to develop *via* a complex interplay between inherent genetic risk and environmental exposures that ultimately trigger autoimmunity (1). While there is a subset of RA/SLE patients that are seronegative, the majority of patients exhibit disease specific autoantibodies (aAb) that can be elevated years prior to the clinical diagnosis of disease during a period that can be termed ‘preclinical autoimmunity’ (2, 3). However, the complete etiology of both RA and SLE remains unknown; in particular, it is not known what factors may drive the development of aAbs during the preclinical period.

Genetic factors are thought to account for 40-50% of RA (4, 5) and 55-77% of SLE (6, 7) risk, leaving approximately half of the risk for disease development unexplained. Epidemiologic studies have identified many environmental factors associated with the risk and severity of disease for both RA and SLE (8, 9). Vitamin D (25-hydoxyvitamin D; 25(OH)D) is one environmental factor that has been studied, since vitamin D deficiency is a common finding in patients who have a clinical diagnosis of an AD including RA and SLE (10). The major role of vitamin D is maintaining normal blood levels of calcium and phosphorus. In addition, 25(OH)D has been shown to have immune-modulatory properties, such as preventing antigen expression, regulating T cell activity and inhibiting cytokine abundance (11–14).

Exposure to natural light is the most common source of vitamin D levels (14–16); and dietary intake of fortified foods or fatty fish is another way in which people gain vitamin D levels (17). However, sunlight exposure and diet can fluctuate throughout an individual’s lifetime, such that a single 25(OH)D measure may not adequately reflect long-term vitamin D status. There is a known genetic influence on 25(OH)D serum levels (18–20). Twin studies have estimated the heritability of vitamin D serum levels to be between 50% and 80% (21, 22). Jiang et al. (19) recently conducted a genome-wide association study (GWAS) on 79,366 individuals of European ancestry and found a select number of single nucleotide polymorphisms (SNPs) that explained 38% of the variance in serum 25(OH)D concentrations. A genetic risk score that predicts vitamin D concentrations (i.e., genetically determined 25(OH)D) may provide a more stable estimate of lifetime vitamin D levels or status.

In this paper, we focus on investigating if genetic determinants of vitamin D levels are inversely associated with autoantibody positivity prior to clinical symptoms in two at-risk populations: first-degree relatives (FDRs) of RA and SLE probands. FDRs of people with an AD are at an increased risk for that AD compared to the general public (23–25). We generated a genetic risk score (GRS) for serum 25(OH)D levels to evaluate the relationship between vitamin D and autoantibody positivity status in at-risk individuals.

## Materials and Methods

### Study Population

We utilized two at-risk cohorts in which we identified RA and SLE probands and their respective unaffected FDRs. Both cohorts have been approved by their institutional review boards (University of Colorado and Oklahoma Medical Research Foundation) and had written informed consent prior to any procedures.

RA FDRs were selected from the Studies of the Etiologies of Rheumatoid Arthritis (SERA), a prospective cohort study that enrolled FDRs of probands with RA (26). RA probands met ≥4 1987 American College of Rheumatology (ACR) RA classification criteria (27). FDRs were tested for rheumatoid factor (RF) isotypes (IgA, IgG, IgM), RF by nephelometry, anti-cyclic citrullinated peptide 2 (CCP2), and/or anti-CCP3.1, as described in James et al. (28). An FDR testing positive for any one of these autoantibodies (aAb) was selected as an aAb+ RA FDR (n = 189). An FDR testing negative for these autoantibodies was selected as an aAb- RA FDR (n = 181). To be consistent with Jiang (19) and reduce confounding due to ethnic and racial difference, all RA FDRs selected for genotyping were non-Hispanic white, and one FDR was randomly chosen from each family so that no FDRs were related to other FDRs (28).

The SLE FDRs were selected from the Lupus Family Registry and Repository (LFRR), a prospective study of FDRs of probands with SLE (29). SLE probands met ≥4 ACR SLE classification criteria (30). FDRs were tested for autoantibodies to Sm, Sm/RNP, RNP, dsDNA, chromatin, ribosomal P, Ro/SSA, La/SSB and/or anti-cardiolipin autoantibodies: IgA, IgG, and IgM, as described in James et al. (28). An FDR testing positive for any one of these aAb was selected as an aAb+ SLE FDR (n=157). An FDR testing negative for these autoantibodies was selected as an aAb- SLE FDR) (n=185). For similar reasons as mentioned above, all SLE FDRs were non-Hispanic white, and one FDR was randomly chosen from each family so that no FDRs were related to other FDRs (28).

### Genotyping & Genetic Risk Score Calculation

RA and SLE FDR DNA samples were genotyped using the Illumina MEGA^EX^ BeadChip and the ImmunoChip v1.0., respectively, per Illumina protocols starting with 250 ng of genomic DNA and read on an Illumina iSCAN. Genome Studio (Illumina) was used for quality control (QC) which included removing SNPs and samples with missing call rates >10%, minor allele frequency < 0·00001, and Hardy Weinberg Equilibrium < 0·001. SNPs that indicated known QC errors (e.g., poor clustering) were also removed.

Jiang et al. (19) identified six SNPs associated with circulating 25(OH)D concentrations in a European ancestry genome-wide association study. Of these SNPs, five SNPs (or their proxies) had been genotyped in the RA FDRs using the MEGA^EX^ BeadChip: rs3755967 (in *GC*, chr4: 71743681), rs12785878 (in *NADSYN1*, chr11:71456403), rs10741657 (in *CYP2R1*, chr 11:14893332), rs10745742 (in *AMDHD1*, chr12:95964751), and rs8018720 (in *SEC23A*, chr14:39086981). For markers rs3755967 and rs10745742, we used proxy SNPs with 100% linkage disequilibrium (LD) according to the 1000 Genomes Project CEU population, whom are Utah residents of Northern and Western European ancestry, and (rs4588 located on chr4:71752606 and rs6538691 located on chr12:95959729, respectively). These five SNPs had not been genotyped with the ImmunoChip, so in order to measure these in the SLE FDR population, we directly genotyped them using the rhAMP™ SNP Genotyping assay (Integrated DNA Technologies) per manufacturers protocols using the forward and reverse primers shown in **Supplemental Table S1**. **Supplemental Table S2** shows details on the markers (and proxy markers) used in the analysis.

To calculate the vitamin D GRS, we summed the number of effect alleles for each of the five markers. For each SNP, an individual would have the potential to have either 0, 1 or 2 effect alleles, leaving the potential GRS of any individual to be an integer between 0 and 10. The effect allele is the allele which was associated with a higher circulating 25(OH)D concentration as reported in Jiang et al. (19). We also dichotomized the vitamin D GRS into high (≥ 5 effect alleles) and low (< 5 effect alleles).

### Statistical Analyses

All analyses were performed within cohort (RA FDRs or SLE FDRs). For genetically determined vitamin D, we tested vitamin D associated SNPs individually under an additive genetic model and the vitamin D GRS as both a continuous and a categorical (high/low) variable. Covariates for further statistical analyses were selected if they were significantly associated (p-value < 0.05) with aAb+ status. A logistic regression was used to identify the genetically determined vitamin D association with autoantibody positivity status while adjusting for sex and age. To address population stratification, we examined ancestry principal components (PCs) that were available for all RA FDRs (using the MEGA^EX^ BeadChip) and for a subset of 304 SLE FDRs (using the ImmunoChip). Because we were concerned about needing to eliminate 59 SLE FDRs from the analyses if we adjusted for the PCs, we performed sensitivity analyses to show that there was no significant change in effect size estimates in both cohorts when the first three ancestry PCs were included in the models (**Supplemental Table S3**). To optimize sample size in the SLE FDRs and keep methods comparable across cohorts, we did not adjust for ancestry PCs in the final statistical models.

## Results

### Demographics of the Study Populations

**Table 1** depicts the demographics of aAb+ and aAb- FDRs in each cohort. In both cohorts, aAb+ FDRs are significantly older than aAb- FDRs; and aAb- FDRs are more likely to be female than aAB+ FDRs.

**Table 1:** Demographic characteristics for the RA FDR and SLE FDR cohorts.

Characteristic	RA aAb+ FDR	RA aAb- FDR	p-value	SLE aAb+ FDR	SLE aAb- FDR	p-value
N	189	181		157	185	
Sex: % female	75.7	86.2	0.01	73.9	83.8	0.03
Age: mean ± SD	51.7 ± 16.2	47.4 ± 15.5	0.01	59.2 ± 15.3	55.7 ± 14.6	0.03
BMI: mean ± SD	26.8 ± 5.5	26.9 ± 6.1	0.88	28.1 ± 5.8	27.3 ± 5.8	0.09
*Ever Smoker: % yes	40.2	41.7	0.78	48.5	48.1	0.91
25(OH)D3 GRS: mean ± SD	4.6 ± 1.4	4.9 ± 1.4	0.03	4.86 ± 1.33	4.67 ± 1.51	0.22
25(OH)D3 GRS High: % yes	49.2	60.2	0.03	42.0	44.3	0.67

*1 SERA FDR missing smoking data.

All patients selected for both cohorts were non-Hispanic white.

### Vitamin D GRS Allele Distribution Across Cohorts

**Figure 1:**
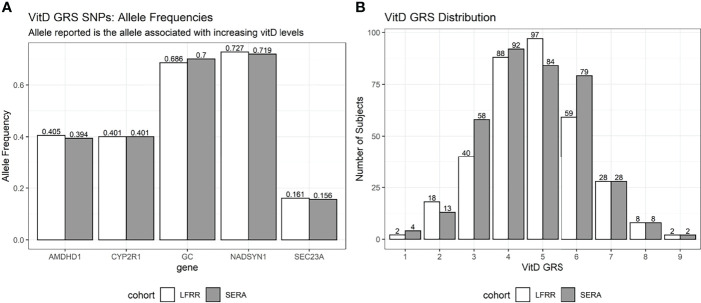
Effect allele frequencies and vitamin D GRS distribution. Effect alleles are those that were associated with an increased 25(OH)D. **(A)** The allele frequency for each of the 5 SNPs used in the vitamin D GRS calculation are shown. The SLE FDRs are shown in white bars (LFRR cohort) and RA FDRs are in gray bars (SERA cohort). **(B)** The distribution of the vitamin D GRS is shown in for each cohort.

The frequencies of the effect alleles of the vitamin D SNPs and the distributions of the vitamin D GRS were similar across the two cohorts (**Figure 1**). The effect allele frequencies ranged from 0.156 and 0.161 for the *SEC23A* SNP to 0.719 and 0.727 for the *NADSYN1* SNP in the RA and SLE FDR cohorts respectively. Both cohorts had a median vitamin D GRS of 5, and a mean (SD) of 4.76 (1.43) and 4.74 (1.45) for RA and SLE FDRs respectively.

### Vitamin D GRS Association With Autoantibody Positivity (aAb+)

**Figure 2:**
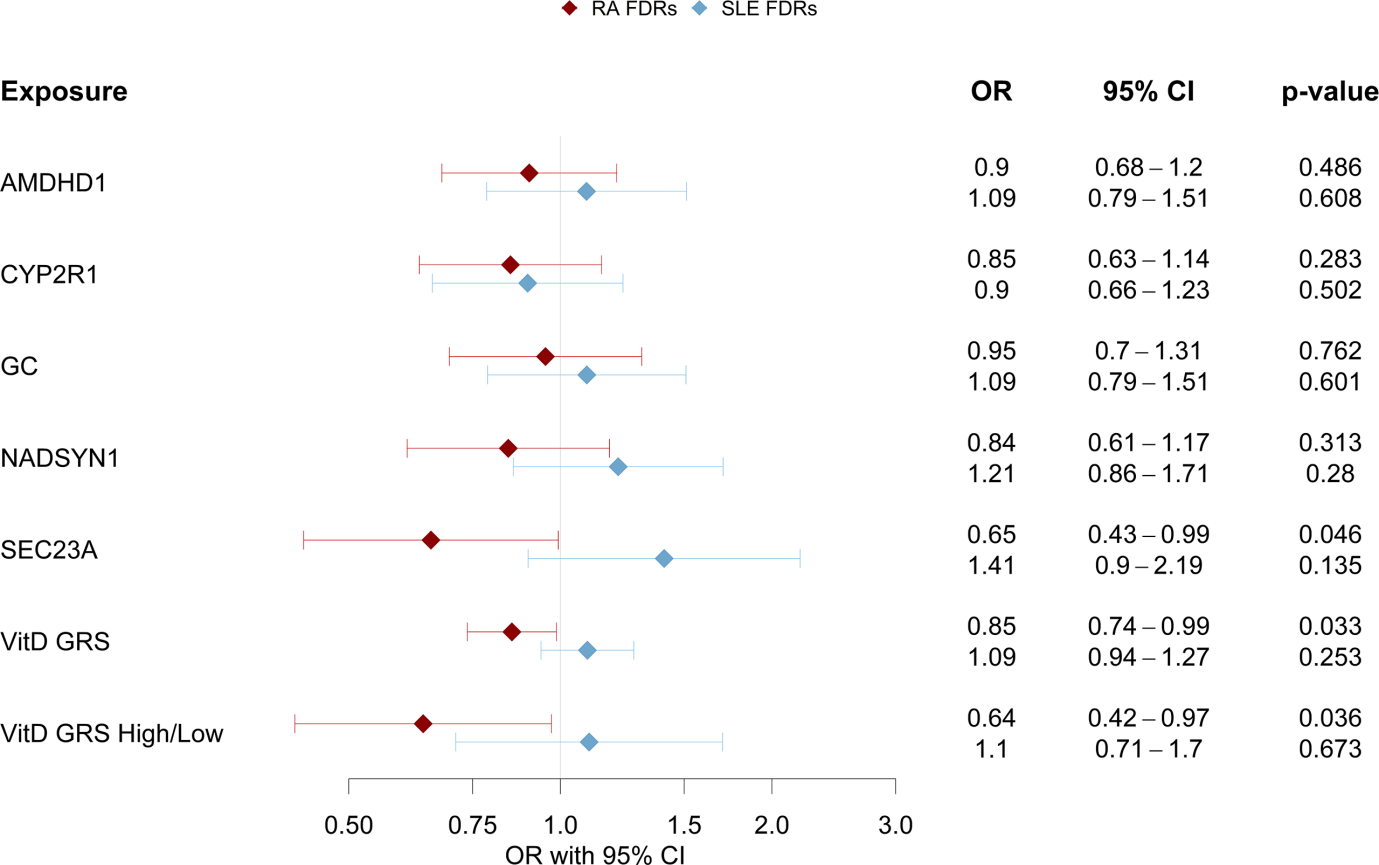
Vitamin D GRS Association with Cohort Specific aAb+. For different vitamin D level measures (either individual SNPs, vitamin D GRS, or dichotomized vitamin D GRS), the odds ratio (OR) is shown as the dot and the corresponding 95% confidence interval is the line. The RA FDR cohort is in red and SLE FDR cohort in blue.

In the SERA RA FDR cohort, the vitamin D GRS (as a continuous variable and as a high/low category) was significantly associated with RA aAb+ status, adjusting for age and sex (**Figure 2**). The presence of a higher number of effect alleles (potentially reflecting a higher lifetime levels of vitamin D) was associated with a lower odds of being aAb+ in the RA FDRs. In addition to the vitamin D GRS, the *SEC23A* was significantly associated with RA aAb+ status in RA FDRs, adjusting for age and sex (OR 0.65; 95% 0.43 to 0.99; p = 0.046). Neither the vitamin D GRS nor any of the vitamin D SNPs were associated with SLE aAb+ status in the SLE FDRs.

### Genetic Risk Score and 25(OH)D Levels: A Sub-Analysis

To investigate whether the vitamin D GRS was associated with 25(OH)D levels, we identified a subset of FDRs in the RA and SLE populations that had had plasma 25(OH)D concentrations measured previously. Twenty-eight of the RA FDRs in the current analysis had plasma 25(OH)D concentration measured previously by radioimmunoassay (DiaSorin, Inc) (31). Sixty-four of the SLE FDRs had plasma 25(OH)D concentrations measured previously using a commercial enzyme immunoassay (Immunodiagnostic Systems, Inc., Scottsdale, AZ) according to manufacturer instructions. To compare 25(OH)D concentrations in high and low vitamin D GRS groups, we performed a Welch’s t-test, which accounts for different variances within the groups. In the RA FDRs, those with a high vitamin D GRS had significantly higher 25(OH)D concentration at a single point in time than those with a low vitamin D GRS {25(OH)D mean [standard deviation (SD)]: 29.3 (2.97) and 24.2 (9.01) ng/mL for high and low GRS, respectively; p-value = 0.04} (**Supplemental Figure S1A**). In SLE FDRs, there was no association between the vitamin D GRS and 25(OH)D concentration [25(OH)D mean (SD): 26.1 (10.2) and 24.3 (8.38) ng/mL for high and low GRS, respectively; p-value = 0.47] (**Supplemental Figure S1B**).

## Discussion

### Association of Vitamin D GRS and RA aAb+ Status

We observed in RA FDRs that a higher vitamin D GRS was associated with lower risk of RA aAb positivity. If indeed the GRS is indicative of longer-term adequate vitamin D levels, this may suggest that long-term adequate vitamin D levels area a possible protective factor for RA aAb+ among individuals at-risk for developing RA. Our results are consistent with the hypothesis that increased 25(OH)D levels may protect again RA through the suppression of cytokines and inflammation (reviewed in (32)). Moreover, supplementation of vitamin D and omega-3 fatty acids was associated with a decreased risk of rheumatoid arthritis in the recently reported VITAL randomized controlled trial (33). Our finding, along with others, suggest that long term vitamin D supplementation may be needed in individuals at-risk for RA, particularly those lacking the effect alleles of SNPs that lead to a higher genetically determined vitamin D level.

### Lack of Association of Vitamin D GRS and SLE aAb+ Status

In contrast, all associations between the vitamin D GRS and the individual SNPs with aAb+ in the SLE FDRs were non-significant. This does not necessarily mean vitamin D levels are not associated with SLE aAb+ but potentially the genetically-regulated component is not associated, or perhaps more complex mechanisms are involved in disease etiology. Young et al. (34) has shown that the relationship between circulating 25(OH)D levels and SLE was modified by a CYP24A1 polymorphism, with each minor allele copy presenting a stronger inverse relationship between 25(OH)D and SLE. Bae and Lee (35) performed a mendelian randomization on vitamin D levels and found no causal association between vitamin D and risk for either RA or SLE. However, this study only assessed SNPs in *SSTR4*, *NADSYN1* and *GC*, and did not examine *SEC23A*, which contained our strongest effect allele.

Additionally, the SLE aAb+ FDRs could possibly be a more heterogenous population than the RA aAb+ FDRs. More autoantibodies were considered for one to be defined as a SLE aAb+ (8 autoantibodies) compared to RA aAb+ (6 autoantibodies). Not only are there various types of autoantibodies for SLE, but it is well noted that patients with SLE have a variety of symptoms occurring in different combinations (31) leading to within-disease heterogeneity (36). This greater heterogeneity may suggest that the vitamin D GRS should be investigated within sub-types of SLE autoimmunity, which requires a larger sample size than that available to the current study.

Interestingly, our vitamin D GRS was not robustly associated with circulating 25(OH)D levels in the SLE FDRs, which may also be an explanation as to why we did not see an association with SLE aAb+ status. And finally, there may be disease-specific effects of vitamin D in AD development. For example, it is possible that the vitamin D GRS is associated with production of RA-related autoantibodies in the preclinical period of RA development as we have observed herein; this may be in contrast to SLE where vitamin D may play a role in the transition from autoantibody positivity to clinical disease onset. Future studies should follow Ab+ individuals for progression to clinical disease to examine this hypothesis.

### SEC23A Role in Immune Response

The *SEC23A* SNP was the only SNP in the vitamin D GRS with a significant protective association with RA aAb+ on its own. This is of interest as the allele represents a missense variant that alters the protein’s amino acid sequence from a leucine to valine and could result in a functional change in the protein. *SEC23A* is a component of the coat protein complex II which is required for the translocation of insulin-induced glucose transporter SLC2A4/GLUT4 to the cell membrane (32). *SEC23A* also has a role in immune function as it is part of the GO Biological Process GO:0002474: antigen processing and presentation of peptide antigen *via* MHC class I. Antigen presentation is a major process in activating both B and T cells, a necessary component for the inflammation process in general (37). In addition, this process has been shown to be important in the pathogenesis of RA (38) and could function differently based on one’s genetic background. Therefore, it is possible that the effect of *SEC23A* on immune function may or may not work through vitamin D levels and requires further exploration.

### Strengths and Limitations

A strength of our study is that we included a large number of at-risk individuals for both RA and SLE; and that these individuals did not have classified disease, which allowed a unique opportunity to examine whether vitamin D SNPs are relevant in the preclinical phase of disease. A limitation of our study is its focus on non-Hispanic whites exclusively, which limits its generalizability. In addition, we only assessed five of the six vitamin D SNPs reported from Jiang (19). Additional genetic markers may be needed to adequately assess the complex relationship of vitamin D and SLE aAb+, as reported by Young et al. (34). Additional limitations include that only a small subset of samples had circulating 25(OH)D levels measured, and that two different 25(OH)D assays were utilized in the two cohorts.

### Conclusion and Future Directions

These findings suggest that a high vitamin D GRS may have a protective role in the development RA-specific autoantibodies in individuals at-risk for RA. We speculate that this may be due to higher lifetime levels of vitamin D or other immune effects of this GRS. Future studies need to expand on the complex role of vitamin D in the preclinical phase of ADs, including assessment of additional vitamin D associated SNPs, longitudinal assessment of 25(OH)D levels, and the study of larger more diverse study populations. An important next step would be to replicate our findings in a more generalizable population. Examining potential modifiable factors for the effect of vitamin D levels (e.g., gene-environment interactions), could lead to new understanding of vitamin D in AD etiology. Finally, as there are an increasing number of prevention studies in pre-clinical RA populations, a therapeutic trial of vitamin D supplementation in this population may be warranted. In addition, we do not have consistently collected vitamin D supplement use across our two populations. And since the point of our GRS analysis was to investigate an estimate of long-term vitamin D levels rather than levels based on current sun exposure (i.e., season), we did not include season of blood draw in our models of RA or SLE aAb outcomes. We note that season of blood draw was not associated with the GRS, so it would not be considered a confounder in the analysis.

## Data Availability Statement

The raw data supporting the conclusions of this article will be made available by the authors, without undue reservation.

## Ethics Statement

The studies involving human participants were reviewed and approved by (SERA cohort) University of Colorado; (LFRR cohort) Oklahoma Medical Research Foundation. The patients/participants provided their written informed consent to participate in this study.

## Author Contributions

LV completed data analysis across both studies and wrote manuscript. EB carried out experiments in SERA, completed data analysis in SERA and contributed to writing manuscript. JS provided patient data and samples in SERA and carried out experiments in SERA. JG, WD, and SM carried out experiments in LFRR. MF carried out experiments in SERA. JK provided patient data and samples in LFRR. KY, MD, TM, JO, MW, JB, RK, PG, CL, KD, JJ, and VH provided experimental, analytical and editorial guidance. JN designed experiments and wrote manuscript. All authors contributed to the article and approved the submitted version.

## Funding

Research reported in this publication was supported by grants from the National Institutes of Health K23AR051461, R01AR051394, U01AI101981, T32AR007534, U19AI082714, P30AR053483, P30GM103510, P30AR073750, UM1AI144292, and U54GM104938.

## Conflict of Interest

The authors declare that the research was conducted in the absence of any commercial or financial relationships that could be construed as a potential conflict of interest.

## Publisher’s Note

All claims expressed in this article are solely those of the authors and do not necessarily represent those of their affiliated organizations, or those of the publisher, the editors and the reviewers. Any product that may be evaluated in this article, or claim that may be made by its manufacturer, is not guaranteed or endorsed by the publisher.
