# Supplementary material for: Relationship Between a Vitamin D Genetic Risk Score and Autoantibodies Among First-Degree Relatives of Probands With Rheumatoid Arthritis and Systemic Lupus Erythematosus
Source: Front Immunol. 2022 Jun 3;13:881332. doi: 10.3389/fimmu.2022.881332 (PMC9205604; doi:10.3389/fimmu.2022.881332)
Supplement: Supplementary file 1 [file DataSheet_1.docx]

Supplementary Material

**Table S1.** Primers used for genotyping SNPs in the SLE FDR population.

| **SNP Assayed** | **Allele Primer 1** | **Allele Primer 2** | **Locus Primer** |
| --- | --- | --- | --- |
| rs4588 | /rhAmp-F/CAGCTTTGCCAGTTCCGrUGGGT/GT2/ | /rhAmp-Y/CAGCTTTGCCAGTTCCTrUGGGT/GT2/ | GCGACTGGCAGAGCGACTAAArAGCAA/GT4/ |
| rs12785878 | /rhAmp-F/TGTCTGATATCACAAAGCTTCGrATCCT/GT3/ | /rhAmp-Y/CTGTCTGATATCACAAAGCTTCTrATCCT/GT3/ | GCCAGCAGACAGGACATGArGGATCA/GT2/ |
| rs10741657 | /rhAmp-F/GGAGATACTTTAGCAGGCArAGGGC/GTl/ | /rhAmp-Y/GGAGATACTHAGCAGGCGrAGGGC/GTl/ | GCCACTTCTTTAGCAGTTGATCTCArGCTC/CGT4/ |
| rs6538691 | /rhAmp-F/TCAGGTAAGGGGACCACAGrGTGGG/GTl/ | /rhAmp-Y/TCAGGTAAGGGGACCACATrGTGGG/GTI/ | GCCAGAGGGCATCCTGGATACrUCCCC/GT3/ |
| rs8018720 | /rhAmp-F/GGGCTCTCTAAAGTACCACrUTACT/GT3/ | /rhAmp-Y/GGGCTCTCTAAAGTACCAGrUTACT/GT3/ | GCACAGAAACGGTCAAATTCTCTTrCATCA/GTl/ |

**Table S2.** SNP markers used to create vitamin D GRS.

| **Gene** | **Chr** | **SNP from Jiang et al (2018) GWAS** | **SNP used** | **Major/Minor Allele** | **Effect Allele** | **Strand Typed** |
| --- | --- | --- | --- | --- | --- | --- |
| GC | 4 | rs3755967 | rs4588* | C/A | C | reverse |
| NADSYN1 | 11 | rs12785878 | Same | C/A | A | reverse |
| CYP2R1 | 11 | rs10741657 | Same | A/G | A | forward |
| AMDHD1 | 12 | rs10745742 | rs6538691* | A/C | A | reverse |
| SEC23A | 14 | rs8018720 | Same | G/C | G | forward |

**Table S3.** Sensitivity Analysis Results. Ancestry PCs were available for a subset of the SLE FDR cohort (n=283) and used for both models in this analysis. For the ancestry adjusted model, a logistic regression was performed to identify the genetically determined vitamin D association with autoantibody positivity status while adjusting for sex, age and the top 3 ancestry PCs. The ancestry non-adjusted model we performed a logistic regression to identify the genetically determined vitamin D association with autoantibody positivity status while adjusting only for sex and age. For both models we calculated the OR for the genetically determined vitamin D variable and calculated the percent change when we adjusted for ancestry. All percent changes were < 10%, indicating the top 3 ancestry PCs were not important covariates(39) in our model.

| **Variable** | **Cohort** | **PC Adjusted OR** | **Not PC Adjusted OR** | **Percent Change** |
| --- | --- | --- | --- | --- |
| AMDHD1 | RA FDRs | 0.912 | 0.899 | -1.50% |
| CYP2R1 | RA FDRs | 0.84 | 0.853 | 1.57% |
| GC | RA FDRs | 0.961 | 0.965 | 0.39% |
| NADSYN1 | RA FDRs | 0.837 | 0.843 | 0.79% |
| SEC23A | RA FDRs | 0.642 | 0.648 | 0.86% |
| VitD GRS | RA FDRs | 0.852 | 0.854 | 0.30% |
| VitD GRS High/Low | RA FDRs | 0.624 | 0.638 | 2.23% |
| AMDHD1 | SLE FDRs | 1.055 | 1.11 | 5.22% |
| CYP2R1 | SLE FDRs | 0.815 | 0.818 | 0.34% |
| GC | SLE FDRs | 1.047 | 1.037 | -0.89% |
| NADSYN1 | SLE FDRs | 1.102 | 1.099 | -0.31% |
| SEC23A | SLE FDRs | 1.475 | 1.502 | 1.80% |
| VitD GRS | SLE FDRs | 1.037 | 1.05 | 1.28% |
| VitD GRS High/Low | SLE FDRs | 0.957 | 1.015 | 6.05% |

**
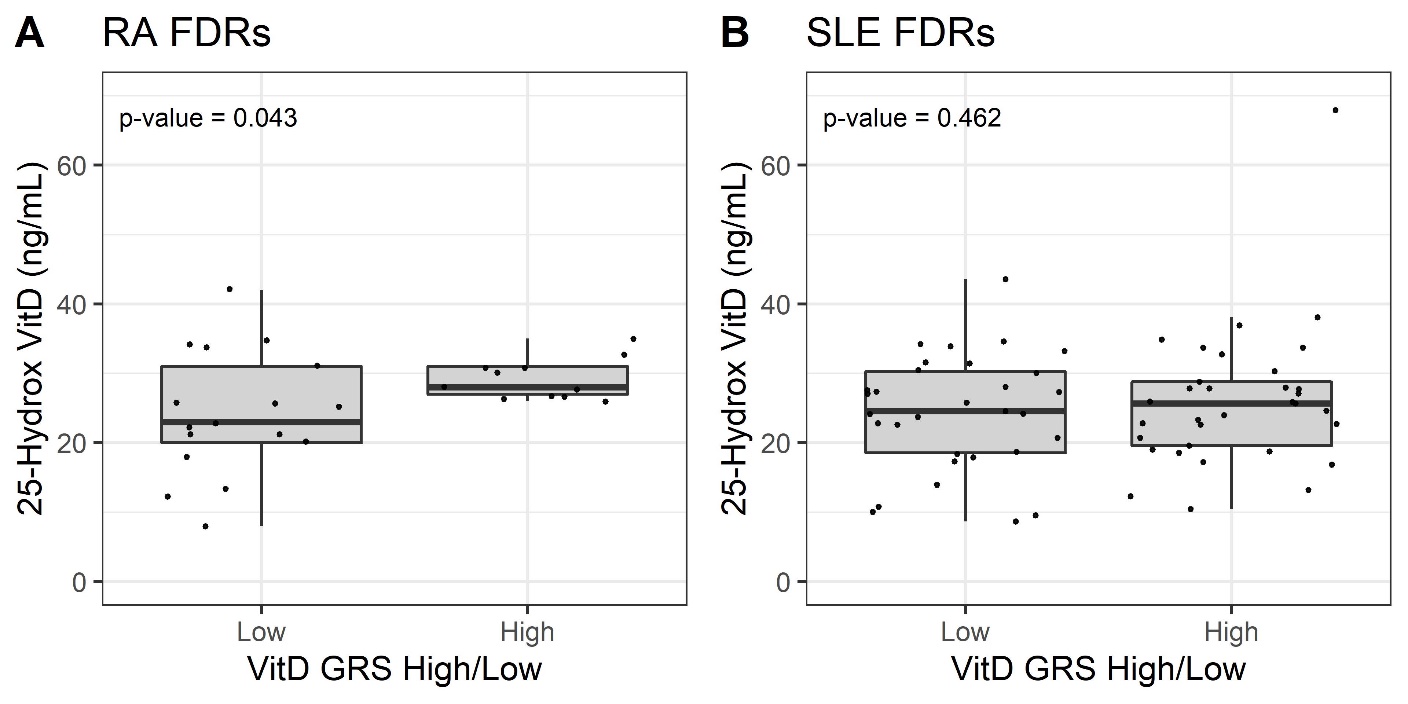
**

**Supplemental Figure S1.** Association of vitamin D GRS and circulating 25(OH)D levels at a single point in time. Boxplots of the measured 25(OH)D levels for the high and low vitamin D GRS groups is shown with summary statistics for each group noted below the boxplots. Low vitamin D GRS is defined with those with < 5 effect alleles, while high vitamin D GRS is defined as those with ≥ 5 effect alleles. A. RA FDRs. B. SLE FDRs.
